# Nanomaterial-Based Molecular Imaging in Cancer: Advances in Simulation and AI Integration

**DOI:** 10.3390/biom15030444

**Published:** 2025-03-20

**Authors:** James C. L. Chow

**Affiliations:** 1 Radiation Medicine Program, Princess Margaret Cancer Centre, University Health Network, Toronto, ON M5G 1X6, Canada; james.chow@uhn.ca; Tel.: +1-416-946-4501; 2 Department of Radiation Oncology, University of Toronto, Toronto, ON M5T 1P5, Canada; 3 Department of Materials Science and Engineering, University of Toronto, Toronto, ON M5S 3E4, Canada

**Keywords:** nanomaterials, nanoparticles, MRI, PET, CT, US, PAI, Monte Carlo simulation, artificial intelligence, machine learning, medical imaging, cancer therapy

## Abstract

Nanomaterials represent an innovation in cancer imaging by offering enhanced contrast, improved targeting capabilities, and multifunctional imaging modalities. Recent advancements in material engineering have enabled the development of nanoparticles tailored for various imaging techniques, including magnetic resonance imaging (MRI), computed tomography (CT), positron emission tomography (PET), and ultrasound (US). These nanoscale agents improve sensitivity and specificity, enabling early cancer detection and precise tumor characterization. Monte Carlo (MC) simulations play a pivotal role in optimizing nanomaterial-based imaging by modeling their interactions with biological tissues, predicting contrast enhancement, and refining dosimetry for radiation-based imaging techniques. These computational methods provide valuable insights into nanoparticle behavior, aiding in the design of more effective imaging agents. Moreover, artificial intelligence (AI) and machine learning (ML) approaches are transforming cancer imaging by enhancing image reconstruction, automating segmentation, and improving diagnostic accuracy. AI-driven models can also optimize MC-based simulations by accelerating data analysis and refining nanoparticle design through predictive modeling. This review explores the latest advancements in nanomaterial-based cancer imaging, highlighting the synergy between nanotechnology, MC simulations, and AI-driven innovations. By integrating these interdisciplinary approaches, future cancer imaging technologies can achieve unprecedented precision, paving the way for more effective diagnostics and personalized treatment strategies.

## 1. Introduction

Cancer imaging plays a critical role in early detection, diagnosis, and treatment planning. Advanced imaging modalities, including computed tomography (CT), magnetic resonance imaging (MRI), positron emission tomography (PET), and ultrasound (US), provide essential anatomical and functional information [1,2]. These techniques enable oncologists to assess tumor progression, monitor therapeutic responses, and guide interventions. However, traditional imaging methods face several challenges, including suboptimal contrast resolution, limited tissue differentiation, and potential risks associated with ionizing radiation. These limitations necessitate the development of novel imaging strategies that enhance sensitivity and specificity while minimizing adverse effects [3,4].

Nanomaterials have emerged as promising agents for improving cancer imaging through enhanced contrast, targeted delivery, and multifunctional capabilities [5]. Their unique physicochemical properties enable selective tumor targeting and increased imaging sensitivity. Quantum dots (QDs), for instance, exhibit strong fluorescence properties, making them suitable for optical imaging [6]. Gold nanoparticles (AuNPs) enhance contrast in CT imaging due to their high X-ray attenuation coefficient [7]. Similarly, superparamagnetic iron oxide nanoparticles (SPIONs) serve as efficient contrast agents for MRI, improving signal intensity and tumor delineation [8]. The integration of nanomaterials in imaging platforms facilitates multimodal imaging, facilitating the combination of different imaging techniques for improved diagnostic accuracy.

**Figure 1:**
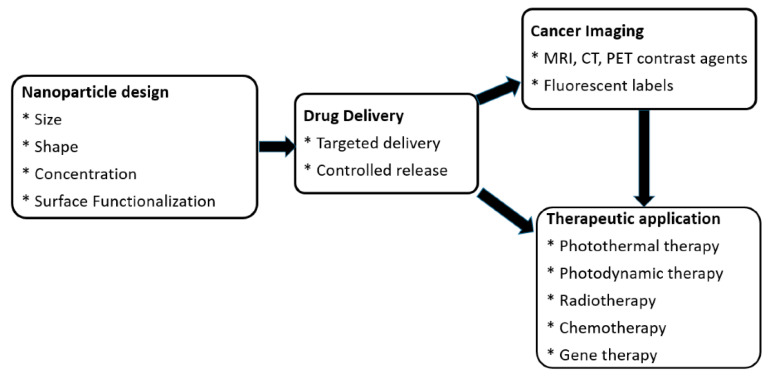
Roles of nanoparticles in nanomedicine for cancer imaging and therapy. Reproduced from reference [9] under the Creative Commons Attribution 4.0 International License (https://creativecommons.org/licenses/by/4.0/ (accessed on 17 March 2025)).

Monte Carlo (MC) simulations are widely used to model the interaction of nanoparticles with biological tissues, providing insights into light propagation, radiation dose distribution, and nanoparticle-mediated contrast enhancement [9]. These simulations enable precise predictions of nanoparticle behavior, optimizing their application in imaging and therapy. Figure 1 highlights the roles of nanoparticles in nanomedicine, particularly in cancer imaging and therapy. It shows how nanoparticle design (size, shape, surface functionalization) enables targeted drug delivery, enhances imaging (MRI, CT, PET, fluorescent labels), and supports therapies like photothermal, photodynamic, radiotherapy, chemotherapy, and gene therapy. AI-driven approaches further enhance cancer imaging by improving image processing, segmentation, and quantitative analysis [10]. Deep learning models are increasingly employed for automated tumor detection, feature extraction, and classification, reducing observer variability and enhancing diagnostic accuracy. AI also supports predictive modeling, enabling early-stage cancer detection and treatment response assessment [11].

This topical review provides a comprehensive analysis of nanomaterial-based imaging, focusing on their applications in various imaging modalities. It examines the role of MC modeling in optimizing nanoparticle interactions and evaluates AI-driven advancements in image analysis and interpretation. By integrating insights from material science, computational modeling, and AI, this review aims to highlight recent innovations and future directions in cancer imaging, emphasizing the potential of nanotechnology-driven approaches in enhancing diagnostic precision and clinical outcomes.

## 2. Nanomaterials in Cancer Imaging

### 2.1. Types of Nanomaterials and Their Applications

#### 2.1.1. Quantum Dots

QDs are semiconductor nanocrystals known for their high fluorescence yield, making them valuable in optical imaging applications. Their broad absorption spectra and narrow emission peaks facilitate the multiplex imaging of tumor markers, enabling simultaneous visualization of multiple molecular targets [12,13]. Moreover, their photostability and high quantum efficiency contribute to enhanced imaging resolution and a prolonged signal duration [14].

#### 2.1.2. Gold Nanoparticles

**Figure 2:**
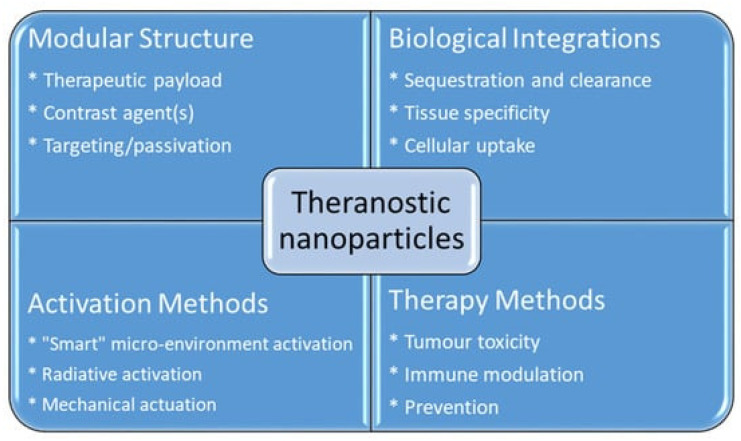
Advantages of using nanoparticles in cancer theranostics. Reproduced from reference [16] under the Creative Commons Attribution 4.0 International License (https://creativecommons.org/licenses/by/4.0/ (accessed on 17 March 2025)).

AuNPs exhibit high X-ray absorption, making them effective contrast agents for CT imaging. Their biocompatibility and ability to be functionalized with targeting ligands facilitate tumor-specific accumulation, improving imaging specificity [15]. Furthermore, AuNPs have been explored in theranostic applications, where they serve both diagnostic and therapeutic purposes, such as in photothermal therapy [16]. Figure 2 shows the advantage of using nanoparticles as a theranostics agent.

#### 2.1.3. Iron Oxide Nanoparticles

Iron oxide nanoparticles, particularly SPIONs, enhance MRI contrast due to their strong magnetic properties. Ferumoxytol, an FDA-approved SPION formulation, has been utilized for imaging lymph nodes and tumor microenvironments [17]. These nanoparticles improve lesion detection and characterization, contributing to more precise diagnosis and treatment planning.

#### 2.1.4. Silica-Based Nanoparticles

Silica-based nanoparticles offer a versatile platform for multimodal imaging. Their biocompatibility and tunable surface chemistry facilitate the incorporation of various imaging probes, including fluorescent dyes, radionuclides, and MRI contrast agents [18]. These nanoparticles enable the integration of multiple imaging modalities, improving diagnostic accuracy and enabling the real-time tracking of therapeutic interventions [19].

#### 2.1.5. Polymeric and Liposomal Nanoparticles

Polymeric and liposomal nanoparticles have been widely investigated for their role in fluorescence and PET imaging. PEGylated liposomes, for instance, have been employed in PET imaging to improve tracer stability and prolong circulation time [20,21]. Their ability to encapsulate imaging agents and therapeutic payloads makes them attractive candidates for combined imaging and drug delivery applications. Table 1 summarizes the unique properties and applications of various nanomaterials in medical imaging and therapy. It highlights the high fluorescence yield of QDs, the biocompatibility and X-ray absorption of AuNPs, and the strong magnetic properties of iron oxide nanoparticles. In addition, it showcases the versatility of silica-based nanoparticles for multimodal imaging and the role of polymeric and liposomal nanoparticles in fluorescence and PET imaging, emphasizing their potential in combined imaging and drug delivery.

### 2.2. Multimodal Imaging Approaches

Multimodal imaging approaches integrate multiple imaging modalities to provide complementary anatomical, functional, and molecular information for improved cancer diagnosis and monitoring [22]. The combination of different imaging techniques overcomes the limitations of individual modalities, enhancing spatial resolution, sensitivity, and specificity. These approaches use the unique advantages of each imaging method, offering a more comprehensive assessment of tumor characteristics, progression, and response to therapy.

#### 2.2.1. MRI-PET Hybrid Imaging with Nanoparticles

A widely explored multimodal imaging strategy is the combination of MRI and PET, facilitated by nanoparticle-based contrast agents. SPIONs conjugated with radiotracers serve as dual-modality contrast agents, enabling high-resolution anatomical imaging from MRI while providing metabolic and functional insights from PET [23,24]. This hybrid imaging modality allows for precise tumor localization and characterization, helping to differentiate malignant from benign lesions. MRI provides superior soft-tissue contrast and structural information, whereas PET detects metabolic activity through radiotracer uptake, improving diagnostic accuracy.

**Figure 3:**
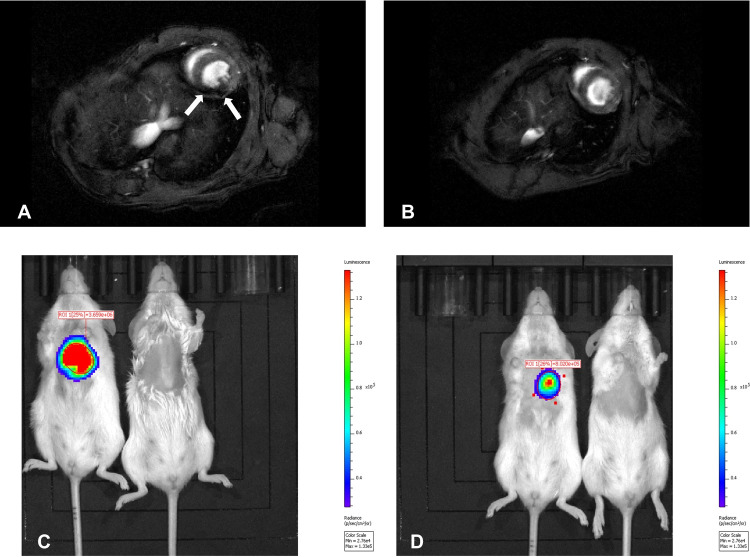
In vivo visualization of firefly luciferase-transduced myoblasts labeled with SPIONs. T2-weighted MR images were obtained from a mouse injected intracardially with SPION-labeled cells (**A**) and a control mouse (**B**). Arrows indicate the hypointense area at the injection site. Bioluminescent imaging was performed 5 (**C**) and 12 (**D**) days post-cell administration, showing distinct bioluminescent activity (left) compared to the control mouse (right). Reproduced from reference [26] under the Creative Commons Attribution 4.0 International License (https://creativecommons.org/licenses/by/4.0/ (accessed on 17 March 2025)).

In preclinical and clinical studies, SPIONs labeled with radionuclides such as Cu-64 [25] and F-18 [26] have demonstrated enhanced imaging capabilities. These hybrid contrast agents improve tumor detection sensitivity, enable the real-time tracking of nanoparticle distribution, and assist in therapy response evaluation. Figure 3 shows the intracardial administration of firefly luciferase-transduced and SPION-labeled myoblasts into the wall of the murine left heart ventricle. This resulted in a distinctly visible hypointense area (Figure 3A) compared to the control mouse without labeled cell transplants (Figure 3B), corresponding to the site of cell administration. Moreover, in vivo bioluminescence imaging of transplanted myoblasts, performed after MRI, showed clear signals from the site of cell administration at 5 (Figure 3C) and 12 days (Figure 3D) post-implantation, with a photon radiance of 3.659 × 10^6^ and 8.02 × 10^5^ photons/sec/cm^2^/steradian, respectively. This indicates that the MR images were obtained from viable SPION-labeled cells.

**Figure 4:**
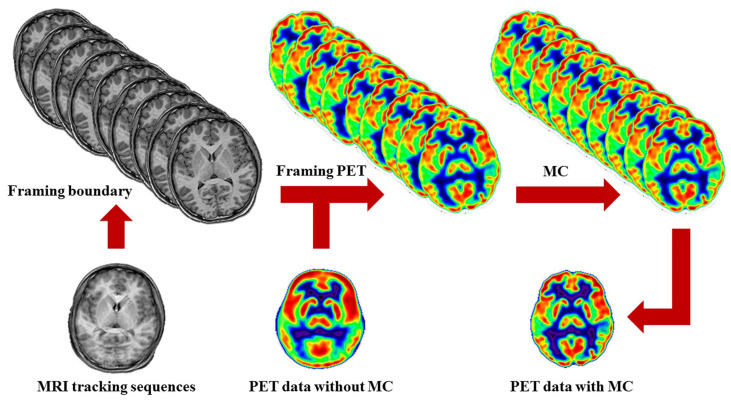
Schematic of MRI-driven motion control procedures. MRI tracking sequences monitor head motion during PET scanning. When head motion exceeds a threshold, it marks actual movement and sets a framing boundary for recombining PET images. This divides PET data into discrete temporal subunits with fixed head poses. This motion control for PET data is completed by assembling these framed PET images into a single PET image. Reproduced from reference [27] under the Creative Commons Attribution 4.0 International License (https://creativecommons.org/licenses/by/4.0/ (accessed on 17 March 2025)).

Moreover, MRI-PET imaging facilitates personalized treatment planning by correlating anatomical and metabolic data, aiding in the assessment of treatment efficacy and early detection of tumor recurrence [27]. Figure 4 shows the MRI-driven motion control procedures. MRI tracking sequences monitor the head motion during PET scanning. When the head motion exceeds a threshold, it marks movement and sets a framing boundary for recombining PET images. This divides PET data into subunits with fixed head poses, which are then assembled into a single PET image.

#### 2.2.2. CT-MRI Dual-Modality Imaging Using Hybrid Nanoparticles

Hybrid nanoparticles incorporating different imaging properties into a single nanostructure have gained significant attention for multimodal imaging applications. A prime example is gold-iron oxide core–shell nanoparticles, which combine the optical and X-ray attenuation properties of gold with the magnetic properties of iron oxide [28]. These nanoparticles function as contrast agents for both CT and MRI, offering high-resolution anatomical imaging from CT and superior soft-tissue contrast from MRI [29].

Gold-coated SPIONs are particularly useful in cancer imaging due to their biocompatibility, tunable surface chemistry, and ability to be functionalized with targeting ligands. These nanoparticles accumulate preferentially in tumors, allowing simultaneous structural and functional imaging [30]. Furthermore, gold-iron oxide hybrids exhibit strong plasmonic resonance, making them suitable for photoacoustic imaging (PAI), which relies on the generation of US waves following light absorption [31]. By integrating MRI, CT, and PAI capabilities, these nanoparticles facilitate the real-time, high-contrast imaging of tumors with enhanced depth penetration and resolution.

#### 2.2.3. US-PAI with Nanoparticle Agents

Another promising multimodal strategy involves combining US and PAI using nanoparticle contrast agents. PAI exploits the photoacoustic effect, where pulsed laser irradiation of nanoparticles generates US waves due to thermal expansion. Gold nanorods, carbon nanotubes, and perfluorocarbon nanodroplets are commonly employed to enhance PAI signals, allowing deep tissue imaging with high optical contrast [32]. The combination of US and PAI provides a noninvasive, radiation-free alternative for tumor detection and vascular imaging [33]. By functionalizing nanoparticles with tumor-specific ligands, researchers have achieved the targeted imaging of cancerous tissues, aiding in early diagnosis and treatment monitoring [34].

#### 2.2.4. Fluorescence-Magnetic Imaging for Guided Surgery

Fluorescence imaging, when combined with MRI, enables precise image-guided surgery by improving tumor delineation and surgical margin assessment [35]. Fluorescent-labeled magnetic nanoparticles, such as SPIONs conjugated with near-infrared (NIR) dyes, serve as dual-modality contrast agents for preoperative MRI and intraoperative fluorescence-guided tumor resection [36]. This approach is particularly beneficial in neurosurgery and breast cancer surgery, where clear tumor margins are critical for complete excision while preserving healthy tissue. The use of fluorescence-MRI hybrid imaging enhances intraoperative visualization, reducing the likelihood of tumor recurrence by ensuring precise tumor removal [37].

### 2.3. Clinical Impact and Future Perspectives

Multimodal imaging is advancing with hybrid nanoparticle formulations that enhance specificity, biocompatibility, and imaging performance. These nanoparticles integrate multiple imaging modalities, such as MRI, PET, and fluorescence imaging, improving tumor visualization and diagnostic accuracy while overcoming limitations like poor tissue penetration and low contrast. Future research focuses on theranostic nanoparticles that combine diagnostic and therapeutic functions, enabling precise imaging alongside targeted drug delivery or photothermal therapy [38,39]. This approach facilitates real-time monitoring and adaptive treatment strategies, potentially revolutionizing cancer care. AI is also transforming multimodal imaging through automated tumor detection, segmentation, and treatment planning [40]. Machine learning (ML) algorithms enhance diagnostic precision, identify subtle patterns, and support personalized therapy decisions by integrating imaging and patient data [41]. By bridging diagnosis and therapy, multimodal nanoparticle-based imaging, combined with AI, offers a powerful tool for precision oncology [42]. These advancements will lead to more accurate diagnoses, improved treatment monitoring, and better patient outcomes in personalized cancer care.

## 3. Monte Carlo Simulations for Nanoparticle-Based Imaging

MC methods are stochastic computational techniques used to simulate complex physical interactions, including photon, electron, and particle transport in biological tissues [43]. These simulations rely on probabilistic models to track individual particle trajectories, providing detailed insights into radiation transport, energy deposition, and tissue interactions [44]. In cancer imaging, MC simulations are instrumental in optimizing imaging parameters, refining contrast agent designs, and assessing radiation dose distributions. Unlike deterministic models, MC techniques account for random scattering, absorption, and secondary radiation effects, making them highly effective for simulating nanoparticle interactions in various imaging modalities [45].

### 3.1. Monte Carlo Simulation in Nanoparticle-Enhanced Imaging

The integration of MC simulations in nanoparticle-based imaging has significantly advanced our ability to model light, X-ray, and magnetic interactions in biological tissues. These simulations allow researchers to predict how different nanomaterials influence imaging contrast, signal enhancement, and therapeutic applications. By accurately modeling the transport and interactions of photons, electrons, and magnetic fields with nanoparticles, MC simulations provide a powerful tool for optimizing imaging systems and improving diagnostic precision [46].

#### 3.1.1. Quantum Dot-Based Optical Imaging

QDs are widely used in optical imaging due to their superior brightness, photostability, and tunable emission spectra. MC simulations are employed to model light propagation in tissues embedded with QDs, enabling the precise estimation of photon scattering, absorption, and fluorescence emission [47,48]. These simulations are crucial for optimizing excitation and emission parameters, enhancing depth penetration, and performing the multiplex imaging of tumor markers. Furthermore, MC models help in the design of biocompatible QDs with tailored optical properties, ensuring their efficient integration into clinical imaging systems for high-sensitivity fluorescence-based tumor detection [49].

#### 3.1.2. Gold Nanoparticle-Enhanced CT Imaging

**Figure 5:**
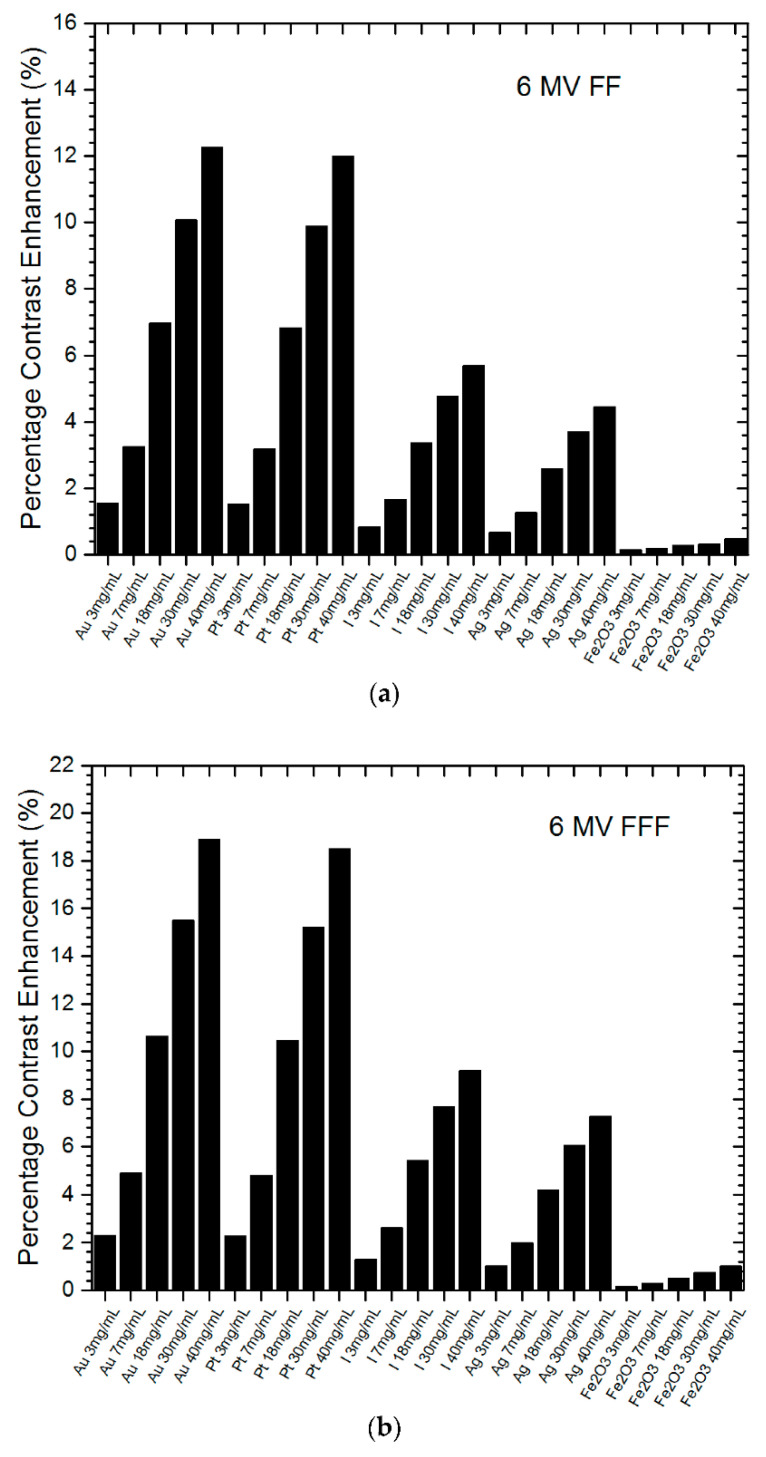
Percentage contrast enhancement for portal imaging with various nanoparticle materials and concentrations using 6 MV (**a**) flattening-filter (FF) and (**b**) flattening-filter-free (FFF) photon beams. Nanoparticles of gold (Au), platinum (Pt), iodine (I), silver (Ag), and iron oxide (Fe_2_O_3_) at concentrations of 3, 7, 18, 30, and 40 mg/mL were used. The percentage contrast enhancement represents the increase in imaging contrast ratio when nanoparticles were added to the tumor. Reproduced from reference [52] under the Creative Commons Attribution 4.0 International License (https://creativecommons.org/licenses/by/4.0/ (accessed on 17 March 2025)).

AuNPs are widely investigated as contrast agents for CT imaging due to their high atomic number (Z = 79) and strong X-ray attenuation properties. MC models play a critical role in simulating how X-ray photons interact with AuNPs in biological tissues, allowing precise predictions of contrast enhancement, energy deposition, and potential radiation dose to surrounding tissues [50]. Key applications of MC simulations in AuNP-enhanced CT and MV portal imaging include optimizing nanoparticle material and concentration, predicting imaging dose deposition, and enhancing image contrast [51,52]. MC-based insights significantly contribute to the development of next-generation AuNP-based contrast agents, facilitating their translation into clinical CT imaging. Figure 5a,b show the percentage contrast enhancement with different nanoparticle materials and concentrations using 6 MV FF and FFF photon beams. The x-axis plots the nanoparticle materials and concentrations (3–40 mg/mL). Zero percentage contrast enhancement indicates no nanoparticles were added. All nanoparticles enhanced portal imaging contrast, with gold and platinum NPs performing the best [52].

#### 3.1.3. SPION-Labeled MRI Contrast Modeling

SPIONs are widely used as contrast agents in MRI due to their strong ability to influence local magnetic fields, thereby enhancing image contrast. MC simulations are critical for modeling the complex interactions between SPIONs and MRI magnetic fields, particularly in estimating changes in transverse relaxation times (T2 and T2*) [53,54]. Applications of MC modeling in SPION-enhanced MRI include assessing magnetic susceptibility effects, guiding SPION design for targeted imaging, and minimizing background signal interference [55]. Through these applications, MC simulations contribute to the refinement of SPION formulations, optimizing their diagnostic capabilities for high-resolution, tumor-specific MRI.

### 3.2. Optimization of Nanoparticle Parameters Using Simulations

MC simulations provide a systematic and quantitative approach for optimizing nanoparticle properties to enhance imaging contrast, biodistribution, and targeting efficiency. By simulating how different parameters influence imaging outcomes, researchers can design nanoparticles with improved diagnostic accuracy and reduced side effects.

#### 3.2.1. Effect of Particle Size and Shape on Imaging Performance

The physical characteristics of nanoparticles—especially their size, shape, and surface properties—significantly impact their interaction with imaging modalities. MC modeling is used to assess the impact of these factors on imaging contrast, biodistribution, and clearance pathways [56]. Size-dependent effects include smaller nanoparticles undergoing rapid renal clearance, medium-sized nanoparticles exhibiting enhanced permeability and retention (EPR) effects, and larger nanoparticles suffering from limited penetration into tumors and prolonged hepatic clearance [57]. Shape-dependent effects involve spherical nanoparticles exhibiting uniform distribution, while rod-shaped or star-shaped nanoparticles have different scattering and absorption properties [58]. MC modeling helps evaluate the orientation-dependent interactions of anisotropic nanoparticles with imaging signals, aiding in the selection of the most effective morphologies for specific applications [59].

#### 3.2.2. Optimizing Concentration and Distribution in Tumor Tissues

Nanoparticle concentration plays a crucial role in imaging contrast and therapeutic effectiveness. Excessive accumulation may lead to signal saturation, toxicity, or undesired immune responses [60], while insufficient concentrations might fail to provide adequate contrast. MC-based studies assist in determining optimal dosing strategies, predicting biodistribution profiles, and reducing off-target effects [61]. By modeling nanoparticle clearance pathways, researchers can refine surface coatings and charge properties to enhance retention at tumor sites while minimizing accumulation in the liver and spleen [62].

#### 3.2.3. Surface Functionalization and Targeting Efficiency

**Figure 6:**
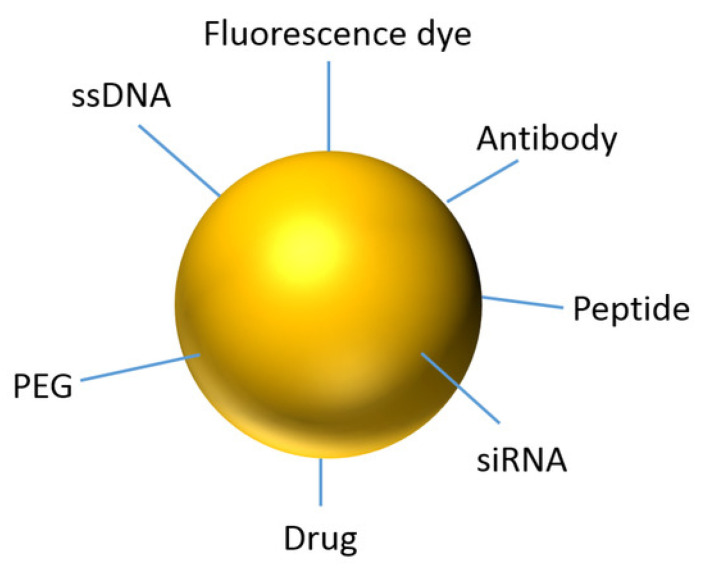
Representation of an AuNP with some possible functional groups. Reproduced from reference [65] under the Creative Commons Attribution 4.0 International License (https://creativecommons.org/licenses/by/4.0/ (accessed on 17 March 2025)).

Functionalized nanoparticles are designed to recognize specific tumor markers, improving imaging specificity and reducing systemic toxicity. MC simulations help evaluate the influence of different surface modifications on nanoparticle behavior. PEGylation effects involve simulations modeling how polyethylene glycol (PEG) coatings influence nanoparticle circulation time, reducing immune system recognition and enhancing tumor uptake [63]. Antibody or ligand conjugation involves MC models predicting how targeting ligands interact with tumor receptors, improving nanoparticle retention and binding efficiency [64]. Charge-dependent targeting involves the surface charge of nanoparticles affecting their interaction with cell membranes. MC simulations assist in fine-tuning charge properties to maximize tumor penetration while preventing non-specific binding to healthy tissues [65]. By using these computational approaches, researchers can rationally design nanoparticles with optimal physicochemical properties, enhancing their efficacy as imaging contrast agents while minimizing adverse effects. Figure 6 shows that various functional groups, including PEG, ssDNA, antibodies, peptides, drugs, fluorescence markers, and siRNA, can be attached to the AuNP surface.

### 3.3. Case Studies and Practical Implementations

MC simulations have been crucial in translating nanoparticle-based imaging concepts into practical applications. These models optimize imaging techniques, evaluate contrast enhancements, and ensure safety in clinical settings. Case studies show their impact on refining nanoparticle-enhanced imaging technologies for diagnostic imaging and therapeutic planning.

**Figure 7:**
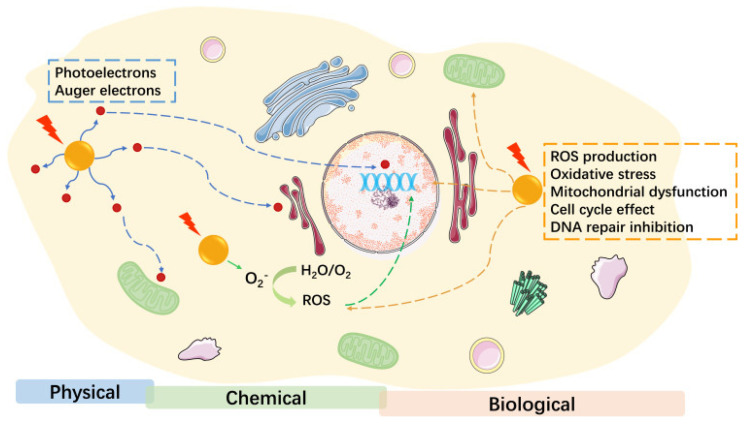
The potential mechanisms of AuNP radiosensitization. Reproduced from reference [66] under the Creative Commons Attribution 3.0 International License (https://creativecommons.org/licenses/by/3.0/ (accessed on 17 March 2025)).

One key application is in nanoparticle-enhanced radiation therapy dosimetry. AuNPs act as radiosensitizers, enhancing localized energy deposition during ionizing radiation [66]. Figure 7 shows the potential mechanisms of AuNP radiosensitization, which can be categorized into three main parts [66]. The first part is physical dose enhancement, which includes mechanisms such as the Compton effect and the photoelectric effect. The second part involves chemical contributions, based on the chemical sensitization of DNA to radiation-induced damage, along with the increased generation and catalysis of radicals. The third part is the biological phase, encompassing various biological mechanisms, including ROS production, oxidative stress, mitochondrial dysfunction, cell-cycle effects, DNA repair inhibition, and other processes such as autophagy and ER stress. These three mechanisms of radiosensitization can be simulated using Monte Carlo MC methods [9].

In CT-guided radiotherapy, AuNPs increase dose deposition in tumors, improving tumor control and reducing radiation exposure to healthy tissues [67]. MC models quantify the dose enhancement factor (DEF) for different nanoparticle concentrations and distributions, providing insights into spatial dose heterogeneity and guiding treatment planning [68]. They also evaluate secondary radiation effects, such as Auger electrons and photoelectrons, enhancing cell killing at the tumor site [69]. Another application is simulating nanoparticle light scattering in optical coherence tomography (OCT). OCT generates high-resolution cross-sectional images based on light scattering in tissues. Nanoparticles like silica or plasmonic gold nanostructures enhance OCT contrast by altering light scattering and absorption in tumor microenvironments [70]. MC-based optical simulations optimize imaging depth, resolution, and contrast, helping design nanoparticle formulations that maximize signal enhancement with minimal biological perturbation [71]. These simulations also assess light penetration depth in highly scattering tissues, crucial for early cancer diagnostics.

MC simulations are also used in other multimodal imaging applications. In fluorescence-guided surgery, they predict fluorescent nanoparticle distribution and emitted light patterns, aiding better tumor margin delineation [72]. In PAI, MC methods optimize nanoparticle compositions for maximum signal generation and biocompatibility [73]. In nanoparticle-enhanced PET/SPECT imaging, MC-based transport simulations evaluate radiation dose distributions from radiolabeled nanoparticles, ensuring safe and effective imaging protocols [74].

Overall, MC simulations play an essential role in advancing nanoparticle-based imaging technologies. They provide predictive insights into imaging performance, contrast optimization, and safety, bridging the gap between theoretical design and practical implementation. As imaging modalities evolve, MC simulations will continue to be a cornerstone of nanoparticle research, aiding the development of next-generation contrast agents and imaging strategies with enhanced precision and clinical utility.

## 4. AI and ML in Nanomaterial-Based Imaging

The integration of AI and ML into nanomaterial-based imaging has innovated the field of medical diagnostics and therapeutic monitoring. AI techniques, particularly deep learning (DL) and reinforcement learning (RL) [75], play a crucial role in enhancing image processing, optimizing nanoparticle-based contrast mechanisms, and improving predictive modeling of nanoparticle–tissue interactions.

### 4.1. AI in Medical Image Analysis

DL techniques have significantly improved the efficiency and accuracy of medical image analysis by enabling automated noise reduction, segmentation, and feature extraction. Convolutional neural networks (CNNs) are widely employed for denoising raw medical images, especially in low-dose CT [76] and MRI [77], enhancing image clarity without compromising diagnostic integrity. Moreover, AI-driven segmentation algorithms facilitate precise delineation of tumors and nanoparticle distributions in hybrid imaging modalities, such as PET/MRI, enabling enhanced detection of malignant lesions with improved specificity and sensitivity [78,79].

ML methods have also been instrumental in tumor detection and classification, particularly in PET/MRI fusion imaging. Hybrid DL models, combining CNNs with recurrent neural networks (RNNs) or transformer-based architectures, have demonstrated superior performance in detecting small tumor masses and differentiating benign from malignant growths [80]. Furthermore, unsupervised learning techniques, including autoencoders and clustering algorithms, provide robust feature extraction and anomaly detection in nanoparticle-enhanced imaging datasets [81].

### 4.2. AI-Driven Nanoparticle-Enhanced Imaging Workflows

The use of AI in nanoparticle-enhanced imaging workflows has led to substantial advancements in automated contrast quantification and biodistribution prediction. AI models, particularly generative adversarial networks (GANs) and attention-based architectures, are capable of extracting quantitative metrics from nanoparticle-enhanced contrast images, ensuring reproducibility and minimizing observer-dependent variability in clinical assessments [82]. These models facilitate automated detection and quantification of contrast uptake in tumor microenvironments, enhancing diagnostic reliability.

DL frameworks have also been developed for predicting nanoparticle biodistribution in vivo [83]. By integrating experimental imaging data with AI-driven predictive models, researchers have improved the accuracy of pharmacokinetic and pharmacodynamic simulations, optimizing nanoparticle formulations for targeted imaging and therapy [84]. Recurrent and graph neural networks (GNNs) are particularly effective in modeling spatiotemporal nanoparticle distribution, offering real-time insights into their interaction with biological tissues [85].

### 4.3. AI-Assisted Monte Carlo Simulations

MC simulations are fundamental for understanding nanoparticle transport and interaction dynamics in biological tissues. AI-driven reinforcement learning approaches have been employed to accelerate MC-based nanoparticle transport modeling, significantly reducing computational time while maintaining simulation accuracy [86]. Reinforcement learning agents optimize particle transport pathways by learning from vast datasets of simulated interactions, enabling the faster and more efficient modeling of nanoparticle behavior in complex tumor microenvironments. In addition, deep neural networks have been used to predict nanoparticle–tissue interaction outcomes, reducing the need for exhaustive MC simulations [87]. By training on high-fidelity MC-generated datasets, DL models can rapidly estimate scattering coefficients, absorption rates, and cellular uptake efficiencies, facilitating real-time decision making in personalized imaging and therapeutic applications. These AI-driven approaches enhance the scalability and applicability of MC simulations in clinical and preclinical research [88].

### 4.4. Future AI Innovations in Cancer Imaging

AI is expected to drive innovations in real-time imaging guidance and nanoparticle synthesis optimization. AI-powered real-time imaging guidance systems are being developed to assist surgeons in intraoperative decision making. These systems employ advanced image processing algorithms, integrated with augmented reality (AR), to provide the dynamic visualization of nanoparticle-enhanced tumor margins, improving surgical precision and minimizing residual disease [89,90]. Moreover, AI is poised to improve nanoparticle synthesis by optimizing formulation parameters for imaging and therapy. ML algorithms, particularly Bayesian optimization and reinforcement learning, are being utilized to streamline nanoparticle design, ensuring optimal contrast properties and biocompatibility [91]. AI-driven synthesis strategies enable rapid screening of potential nanoparticle candidates, accelerating the translation of novel imaging agents from bench to bedside [92]. To sum up, AI and ML have profoundly transformed nanomaterial-based imaging, enhancing diagnostic accuracy, improving simulation efficiency, and paving the way for next-generation imaging innovations. As AI technologies continue to evolve, their integration with nanomedicine will further refine imaging workflows and expand the possibilities for personalized cancer diagnostics and therapeutics [93].

## 5. Clinical Translation and Future Directions

The successful clinical translation of nanomaterial-based imaging technologies is contingent upon their safety, regulatory approval, and integration into existing medical workflows. While numerous nanomaterial-based contrast agents have demonstrated promising results in preclinical and early clinical studies, challenges remain in bridging computational advancements with real-world clinical applications.

### 5.1. Current Status of Nanomaterials in Clinical Imaging

Several nanoparticle-based imaging agents have advanced to clinical trials, demonstrating their potential for enhanced diagnostic precision. Ferumoxytol, an iron oxide nanoparticle, has been employed in MRI for iron-sensitive imaging, enabling improved visualization of inflammatory and vascular structures [94,95]. Furthermore, AuNP-based contrast agents are undergoing preclinical and early clinical evaluation for their superior X-ray attenuation properties, offering potential advantages in CT and multi-modal imaging applications [96,97]. Despite these advancements, widespread clinical adoption remains limited due to challenges such as biodistribution variability, long-term biocompatibility, and large-scale manufacturing constraints. Ongoing research aims to refine nanoparticle formulations to enhance stability, reduce systemic toxicity, and improve targeted delivery to pathological tissues [98].

### 5.2. Bridging the Gap Between Computational Models and Clinical Use

To facilitate the clinical translation of nanomaterial-based imaging, efforts are being made to integrate MC-based simulation data into clinical imaging software. By incorporating high-fidelity simulation outputs into imaging platforms, clinicians can better predict nanoparticle behavior, improving diagnostic accuracy and therapeutic planning [99,100,101]. Moreover, AI-driven personalized imaging protocols are being developed to optimize nanoparticle administration based on individual patient characteristics [102]. ML algorithms can analyze patient-specific parameters to tailor imaging protocols, ensuring optimal contrast enhancement while minimizing unnecessary exposure to nanoparticles.

### 5.3. Regulatory and Safety Considerations

Regulatory approval is a critical step in the clinical adoption of nanomaterial-based imaging agents. The U.S. Food and Drug Administration (FDA) and the European Medicines Agency (EMA) have established guidelines for evaluating the safety and efficacy of nanoparticle-based contrast agents [103]. Key considerations include pharmacokinetics, long-term retention, and potential immunogenicity. To address safety concerns, strategies for toxicity mitigation and long-term monitoring are being actively explored. Biodegradable nanoparticles, functionalized coatings, and controlled release mechanisms are being developed to enhance safety profiles [104]. Furthermore, longitudinal studies and post-market surveillance are essential to monitor long-term outcomes and ensure continued patient safety.

While nanomaterial-based imaging technologies hold immense promise, their clinical translation requires multidisciplinary collaboration, rigorous validation, and regulatory compliance [105,106]. By bridging the gap between computational advancements and clinical implementation, these technologies have the potential to revolutionize medical imaging and personalized diagnostics. Table 2 outlines key factors for clinical translation of nanomaterial-based imaging technologies. It highlights the status of imaging agents like Ferumoxytol in MRI and gold nanoparticles in preclinical studies, computational advancements, and regulatory and safety considerations. Addressing these factors aids in integrating these technologies into routine medical practice. In addition, Table 3 highlights AI’s role in medical imaging, radiotherapy planning, nanoparticle imaging, personalized medicine, and regulatory processes, emphasizing its impact on improving diagnostic accuracy, treatment planning, and safety assessments.

## 6. Conclusions

Nanomaterial-based imaging has emerged as a transformative approach in cancer diagnostics, offering enhanced contrast, molecular specificity, and multimodal capabilities. MC simulations have played a critical role in optimizing imaging contrast and predicting nanoparticle behavior, while AI has significantly improved image processing, segmentation, and simulation efficiency. The integration of these computational tools has led to substantial advancements in precision imaging.

Future progress in cancer imaging will require continued interdisciplinary research at the intersection of material science, computational physics, and artificial intelligence. The development of AI-driven, nanoparticle-enhanced imaging systems holds great promise for advancing precision medicine, enabling real-time guidance in clinical settings, and tailoring imaging protocols to individual patient characteristics. By fostering collaboration across disciplines, these innovations have the potential to redefine cancer diagnostics and treatment strategies, ultimately improving patient outcomes.

## Figures and Tables

**Table 1:** Summary of types of nanomaterials and their applications.

Type of Nanomaterial	Applications
Quantum Dots (QDs) [12,13,14]	- High fluorescence yield for optical imaging; - Broad absorption spectra and narrow emission peaks for multiplex imaging of tumor markers; - Photostability and high quantum efficiency for enhanced imaging resolution.
Gold Nanoparticles (AuNPs) [15,16]	- High X-ray absorption for CT imaging; - Biocompatibility and functionalization with targeting ligands for tumor-specific accumulation; - Theranostic applications, including photothermal therapy.
Iron Oxide Nanoparticles [17]	- Enhance MRI contrast due to strong magnetic properties; - Ferumoxytol for imaging lymph nodes and tumor microenvironments; - Improve lesion detection and characterization for precise diagnosis and treatment planning.
Silica-Based Nanoparticles [18,19]	- Versatile platform for multimodal imaging; - Biocompatibility and tunable surface chemistry for incorporating various imaging probes; - Integration of multiple imaging modalities for improved diagnostic accuracy.
Polymeric and Liposomal NPs [20,21]	- Role in fluorescence and PET imaging; - PEGylated liposomes for improved tracer stability and prolonged circulation time; - Encapsulation of imaging agents and therapeutic payloads for combined imaging and drug delivery.

**Table 2:** Overview of clinical translation of nanomaterial-based imaging technologies.

Category	Key Aspects	Current Status
Nanomaterials in Clinical Imaging [94,95,96,97,98]	Ferumoxytol (iron oxide NPs) for MRI	Approved for off-label use
	Au nanoparticles for X-ray contrast in CT and multi-modal imaging	Preclinical and early clinical trials
Bridging Computational Models and Clinical Use [99,100,101,102]	MC simulation integration	Incorporating high-fidelity simulation outputs into clinical imaging platforms
	AI-driven personalized imaging protocols	Using ML to tailor imaging protocols based on patient-specific parameters
Regulatory and Safety Considerations [103,104,105,106]	FDA and EMA guidelines	Evaluation criteria include pharmacokinetics, long-term retention, and immunogenicity
	Toxicity mitigation strategies	Development of biodegradable nanoparticles, functionalized coatings, and controlled release mechanisms
	Long-term monitoring	Implementation of longitudinal studies and post-market surveillance

**Table 3:** Summary of current clinical applications of AI and potential developing applications in nanomaterial-based imaging and therapy.

Application Area	Current Clinical Applications	Potential Developing Applications	References
Medical Imaging	AI-assisted image interpretation in radiology (e.g., detecting lung nodules in CT, breast cancer in mammography)	AI-driven real-time image enhancement, AI-automated image segmentation for nanoparticle tracking	[94,95,96,97,98]
Radiotherapy Planning	AI-based auto-contouring of organs-at-risk, dose prediction models	Quantum computing-enhanced AI for Monte Carlo-based treatment planning	[99,100,101]
Nanoparticle Imaging	AI-driven segmentation of nanoparticle contrast agents in MRI and CT	AI-predictive modeling of nanoparticle biodistribution and pharmacokinetics	[102,103]
Personalized Medicine	AI-assisted patient stratification for targeted therapies	AI-integrated multi-modal imaging and omics data fusion for individualized treatment planning	[102,103,104]
Regulatory and Safety	AI-supported quality control in nanoparticle manufacturing	AI-guided risk assessment and regulatory decision-making automation	[105,106]

## Data Availability

No new data were created.

## Abbreviations

The following abbreviations are used in this manuscript:

MRI	Magnetic resonance imaging
CT	Computed tomography
PET	Positron emission tomography
US	Ultrasound
MC	Monte Carlo
AI	Artificial intelligence
ML	Machine learning
QDs	Quantum dots
AuNPs	Gold nanoparticles
SPIONs	Superparamagnetic iron oxide nanoparticles
PAI	Photoacoustic imaging
NIR	Near-infrared
OCT	Optical coherence tomography
DL	Deep learning
RL	Reinforcement learning
CNNs	Convolutional neural networks
RNNs	Recurrent neural networks
GANs	Generative adversarial networks
GNNs	Graph neural networks
AR	Augmented reality
FDA	Food and drug administration
EMA	European medicines agency
